# A Case-Control Study Utilizing Red Cell Distribution Width as a Bio-Inflammatory Marker in Pre-eclampsia

**DOI:** 10.7759/cureus.55910

**Published:** 2024-03-10

**Authors:** Khushboo Singhal, Shweta Gupta, Sunita Tiwari, Mohammed Jaffer Pinjar

**Affiliations:** 1 Department of Physiology, Subharti Medical College, Meerut, IND; 2 Department of Physiology, Prasad Institute of Medical Sciences, Lucknow, IND; 3 Department of Physiology, Dr. Ram Manohar Lohia Institute of Medical Sciences, Lucknow, IND; 4 Department of Physiology, All India Institute of Medical Sciences, Deoghar, Deoghar, IND

**Keywords:** hematological analyzer, biomarker, pregnancy, preeclampsia, red cell distribution width

## Abstract

Introduction: This research was conducted to assess the effectiveness of red cell distribution width (RDW) as an indicator for pre-eclampsia (PE), a condition characterized by elevated blood pressure and the presence of protein in the urine occurring beyond the 20th week of pregnancy.

Methodology: The case-control investigation spanned 10 months, following the acquisition of informed consent and the receipt of ethical clearance. The study sample comprised a total of 70 pregnant women, evenly divided into two groups: 35 cases of PE and 35 normotensive pregnant controls. Both the cases and controls provided 3 ml venous blood samples. The study employed a semi-automated three-part hematological analyzer to establish the baseline RDW for all individuals.

Results: This study showed that the individuals with pre-eclampsia had a greater RDW compared to the healthy pregnant women. The observed difference was found to be statistically significant, with a p-value of 0.004. The receiver operating curve (ROC) analysis showed that RDW exhibited significant diagnostic accuracy in differentiating between cases and controls (area under the curve [AUC] = 0.71, P = 0.004) when employing a cut-off value of >= 18.25. The sensitivity was 80% and the specificity was 71.4%.

Conclusion: In contrast to other indicators of inflammation, RDW is a cost-effective and easily accessible biomarker that can be acquired from routine complete blood counts. It has the potential to be valuable in predicting and diagnosing pre-eclampsia.

## Introduction

Preeclampsia (PE), a pregnancy-specific systemic ailment, impacts 2-4% of expectant mothers. The disease is a significant contributor to maternal illness and death [1]. PE is thought to be initiated by multiple causes, such as inflammation, dysfunction of the endothelium, angiogenesis, abnormal placental development, oxidative stress, and immunological and genetic factors [2]. The clinical symptoms of PE are linked to widespread malfunction of the endothelium, resulting in vasoconstriction and reduced blood flow to organs, leading to numerous abnormalities in blood composition [3,4]. Typically, they appear as minor disorders related to blood clotting, such as the consumption of platelets and clotting components. Immune system modifications are pivotal in the onset of PE [5].

The red cell distribution width (RDW) is a hematological parameter that reflects the variability in red blood cell size, commonly referred to as anisocytosis [6-8]. Traditionally utilized for distinguishing types of anemia, RDW has been increasingly associated with hypertension and a range of other cardiovascular risk factors in recent times [9]. The precise mechanism underlying this correlation remains unknown; nevertheless, elevated RDW levels are thought to be indicative of heightened inflammation [9]. Although the link between RDW and hypertension is well-established [9], information on RDW levels in PE patients remains sparse. Therefore, this research aimed to evaluate RDW in pregnant women with PE compared to those without, given the inconsistent data.

## Materials and methods

This study was a 10-month case-control conducted at the physiology, pathology, and obstetrics and gynecology departments at King George’s Medical University in Lucknow, India. The research took place between November 2017 and August 2018 after obtaining written informed consent and the Institutional Ethics Committee's endorsement. The study population consisted of pregnant females aged 18 years and above who reported to the obstetrics and gynaecology department. Among the 70 participants, 35 were identified as having PE based on the updated standards set by the American College of Obstetrics and Gynecology in 2013. The study did not include patients with anaemia, multiple gestations, polyhydramnios, infectious diseases diagnosed during pregnancy, pre-pregnancy hypertension, premature rupture of membrane, renal diseases, diabetes mellitus, inflammatory diseases, or indicators of additional coexisting medical issues that could impact the maternal RDW values. The control group consisted of normotensive pregnant women carrying a single fetus, matched for gestational age, who did not exhibit any indications of prenatal complications or fetal distress. There was an absence of hypertension or proteinuria, and they successfully gave birth to healthy newborns with a size that was adequate for their gestational age. Both the cases and controls were selected by a simple random method. Following an eight-hour fast, a sample of 3 mL of venous blood was collected and divided into two equal portions. A specimen was gathered in a standard vial to assess the lipid panel after fasting. The remaining portion was taken in a vial containing EDTA to evaluate the complete blood count. Pre-birth blood samples were collected from all subjects by puncturing the antecubital vein. A semi-automated three-part hematology analyzer was employed to do a comprehensive blood count, which provided measures for RDW, hemoglobin, white blood cell count, and various other hematological indices [8].

Quantitative analysis

The information was processed with SPSS v. 21.0, designed for statistical computations (IBM Corp., Armonk, NY). The results were presented as number (percentage) and mean ± standard deviation. Statistical computations were performed to contrast the RDW levels between the groups with PE and the normotensive controls. The receiver operating curve (ROC) analysis was employed to assess the optimal threshold values of RDW for distinguishing between the case and control groups. Calculating the area under the curve was essential to determine the best balance between sensitivity and specificity for the test to be significant. Furthermore, the optimal RDW cut-off point was used to determine the sensitivity, specificity, as well as positive and negative predictive values. A p-value below 0.05 was deemed statistically significant.

## Results

The ages of the participants ranged between 20 to 35 years. The majority of women in both categories were between the ages of 26 and 30. Each group consisted of 14 women who accounted for 40% of the total and were 25 years and under. In the case group, there were two women over the age of 30, which accounted for 5.7% of the group; there were no women over 30 in the control group. The average age of cases was 26.31 years with a standard deviation of 3.36, while the average age of controls was 25.91 years with a standard deviation of 2.32. There was no statistically significant difference in age between the two groups (p=0.350) (Table 1). The majority of participants (n=32; 45.7%) had a gestational age of 32-37 weeks, followed by those with a gestational age greater than 37 weeks (n=31; 44.3%), while the fewest had a gestational age of 28-32 weeks (n=7; 10%). The percentage of cases with a gestational age of 32-37 weeks was higher (48.6%) compared to controls (42.9%), while the percentage of cases with a gestational age of 28-32 weeks and over 37 weeks (8.6% and 42.9% respectively) was lower than controls (11.4% and 45.7% respectively). The statistical analysis showed that there was no significant difference between the two groups (p=0.861) (Table 2).

**Table 1:** Comparison of age between the groups Chi-square=2.100; p=0.350

SN	Age Group (years)	Cases (n=35)		Controls (n=35)		Total (N=70)	
		No.	%	No.	%	No.	%
1-	Upto 25	14	40.0	14	40.0	28	40.0
2-	26-30	19	54.3	21	60.0	40	57.1
3-	>30	2	5.7	0	0.0	2	2.9
	Range	20-35		20-29		20-35	
	Mean±SD	26.31±3.36		25.91±2.32		26.11±2.87	

**Table 2:** Comparison of gestational age between the groups Chi-square=0.300; p=0.861

SN	Gestational Age (Weeks)	Cases (n=35)		Controls (n=35)		Total (N=70)	
		No.	%	No.	%	No.	%
1-	28-32	3	8.6	4	11.4	7	10.0
2-	32-37	17	48.6	15	42.9	32	45.7
3-	>37	15	42.9	16	45.7	31	44.3

As anticipated, patients with preeclampsia had markedly increased diastolic and systolic blood pressure (p<0.001), along with elevated amounts of urine protein, in comparison to the healthy control group (p<0.001). An analysis was conducted on the anthropometric traits, which encompassed height, weight, and BMI. The statistical analysis revealed no significant difference between the two groups with these characteristics (p=0.399, 0.371, and 0.068) (Table 3). The mean hemoglobin readings were examined and no statistically significant difference was observed across the groups (p>0.295). The average value of red cell distribution width in mothers was measured and compared. The difference was found to be substantially higher in cases compared to controls (19.49±2.85 versus 17.11±3.71; p=0.004) (Table 4). The receiver operating characteristic (ROC) study revealed that the red cell distribution width (RDW) exhibited significant diagnostic precision in differentiating between cases and controls (area under the curve [AUC] = 0.71) when employing a threshold of >= 18.25. The RDW exhibited 80% sensitivity and 71.4% specificity, as shown in Tables 5-6.

**Table 3:** Comparison of anthropometric parameters between the groups BMI: body mass index; SD: standard deviation

SN	Anthropometric Variables	Cases (n=35)		Controls (n=35)		Statistical significance	
		Mean	SD	Mean	SD	‘t’	‘p’
1-	Height (cm)	156.94	3.56	157.69	3.76	0.849	0.399
2-	Weight (kg)	58.37	4.80	57.49	3.30	0.900	0.371
3-	BMI (kg/m^2^)	23.67	1.28	23.12	1.08	1.931	0.068

**Table 4:** Comparison of hematological parameters between the groups Hb: hemoglobin; RDW: red cell distribution width

SN	Variables	Cases (n=35)		Controls (n=35)		Total (N=70)	
		Mean	SD	Mean	SD	‘t’	‘p’
1-	Hb (gm/dL)	12.41	1.09	12.67	0.93	-1.054	0.295
2-	RDW(%)	19.49	2.85	17.11	3.71	3.016	0.004

**Table 5:** ROC analysis for the area under the curve ROC: receiver operating curve; SE: standard error

Test Result Variable(s)	Area	SE	‘p’	Asymptotic 95% Confidence Interval	
				Lower Bound	Upper Bound
RDW	0.711	0.066	0.002	0.581	0.841

**Table 6:** Forecasted threshold values for various metrics and their respective diagnostic effectiveness PPV: positive predictive value; NPV: negative predictive value

SN	Parameter	Cut-off value	Sensitivity	Specificity	PPV	NPV	Accuracy
1-	RDW	>18.25	80.0	71.4	71.8	77.4	74.3

## Discussion

PE is a condition characterized by high blood pressure and dysfunction of multiple organ systems, the exact cause of which is still unknown. The RDW, traditionally employed to differentiate anemia, has been discovered to be linked to inflammation and oxidative stress [9]. Recent studies have also utilized RDW as a prognostic indicator for morbidity and mortality in many diseases, particularly in cardiovascular disorders [7-10]. The present study utilized ROC analysis to establish the diagnostic accuracy for predicting PE. The study revealed an approximate sensitivity of 80% and specificity of 71.4% when using a cut-off value of 18.25 to differentiate between instances of preeclampsia and healthy pregnant controls.

In line with our findings, Kurt et al. [8] demonstrated a substantial elevation in RDW levels in patients with severe PE compared to healthy pregnant controls and moderate PE (14.1±1.1 vs 16.9±1.7). Furthermore, this rise in RDW levels was observed in both the presence and severity of PE. In a study conducted by Abdullahi et al. [11], it was observed that there were conflicting results about the correlation between RDW and PE among pregnant Sudanese women. The study found that the difference in RDW levels between the two groups was not statistically significant, with the first group having an average RDW of 14.5% with a standard deviation of 1.8% and the second group having an average of 14.4% with a standard deviation of 1.4% (p-value = 0.710). Multiple potential processes can elucidate the correlation between RDW and PE.

It is still uncertain whether anisocytosis (as measured by RDW) is a direct, primary cause of certain conditions, or if it is merely a secondary effect or symptom of an underlying condition, or potentially a result of both primary causation and secondary effects. The most likely underlying mechanism is the heightened inflammatory response, which leads to elevated RDW levels by interfering with the erythropoietin and iron metabolism response, as well as reducing the lifespan of red blood cells [12]. Moreover, it has been demonstrated that inflammatory cytokines hinder the maturation of erythrocytes, resulting in the entry of immature erythrocytes into the bloodstream [13]. Troeger et al. found that patients with PE showed an increase in erythropoietic activation, which was linked to placental hypoxia [14]. Due to their limited regenerative capacity, erythrocytes are susceptible to destruction from even minor incidents that result in damage. In the case of PE, an intensified inflammatory reaction that includes neutrophils, monocytes, and macrophages leads to the breakdown of red blood cells due to their exposure to oxygen radicals and proteolytic enzymes. Following this breakdown, an increase in the plasma levels of certain degradation products, like the erythrocyte membrane band 3 protein, has been documented [15-17]. Tissue hypoxia triggers the secretion of erythropoietin, which in turn stimulates bone marrow. As a result, the number of immature red blood cells circulating in the bloodstream substantially increases, resulting in reticulocytosis (the presence of a high number of reticulocytes (immature red blood cells) in the blood [18].

It is our view that the initiation of inflammatory processes, which are thought to have a strong connection to PE, increases RDW values by influencing red blood cell synthesis. Furthermore, anisocytosis and elevated RDW may be influenced by oxidative stress and damage, in addition to inflammation. The RDW marker, routinely included in a complete blood count, is an affordable measurement tool. It has the potential to provide valuable diagnostic and prognostic information in patients with preeclampsia, similar to other cardiovascular disorders. Overall, incorporating regular RDW measurements during antenatal follow-up can be beneficial for identifying high-risk pregnancies associated with PE [19].

The study has limitations due to its small sample size and single-center methodology, which could impact the generalizability of the findings. Comparison between gravidas suffering from PE across various trimesters was not performed due to limited data availability. Nevertheless, conducting extensive cohort studies is necessary to gain a comprehensive understanding of the pathophysiologic connection between RDW and PE, as well as its causative role. The ultimate goal is to initiate preventive treatment in women affected by PE, as it is a pregnancy complication that poses significant risks to both the mother and the fetus.

## Conclusions

The findings of this study would assist us in determining the role of RDW as a biomarker for inflammation in patients with severe PE. This knowledge would be valuable for implementing preventive healthcare measures and identifying individuals suffering from severe PE and subsequent cardiovascular disorders. Periodically assessing RDW during prenatal surveillance can be advantageous in predicting high-risk pregnancies for PE. RDW can function as a prognostic and diagnostic tool for PE in patients with concealed hypertension, unlike numerous other markers of inflammation and bioassays. The primary benefit is its cost-effectiveness, affordability, and accessibility through a regularly conducted complete blood count.
